# Site-specific coupling between aortic pulse wave velocity, carotid vessel wall thickness and peripheral stenosis severity in peripheral arterial occlusive disease at 3T MRI

**DOI:** 10.1186/1532-429X-15-S1-P247

**Published:** 2013-01-30

**Authors:** Harrie van den Bosch, Jos J Westenberg, Lucien E Duijm, Alette Daniels-Gooszen, Gwat Yoe Mireille The, Joep A Teijink, Albert de Roos

**Affiliations:** 1 Radiology, Catharina Hospital, Eindhoven, Netherlands; 2 Radiology, Leiden University Medical Center, Leiden, Netherlands; 3 Vascular Surgery, Catharina Hospital, Eindhoven, Netherlands

## Background

Arterial wall stiffening and thickening are part of the atherosclerosis process leading to luminal narrowing. A close link between distal aortic pulse wave velocity (PWV) and peripheral vascular disease, reflecting site-specific relationship between local plaque burden and vascular disease, is hypothesized. Purpose of this study was to compare peripheral arterial stenosis severity on contrast-enhanced MR angiography (CE-MRA) with regional aortic wall stiffness, expressed by PWV and acquired with velocity-encoded (VE) MRI, and vessel wall (VW) thickness sampled in the left common carotid artery with 3T Black-Blood (BB) MRI.

## Methods

**Figure 1:**
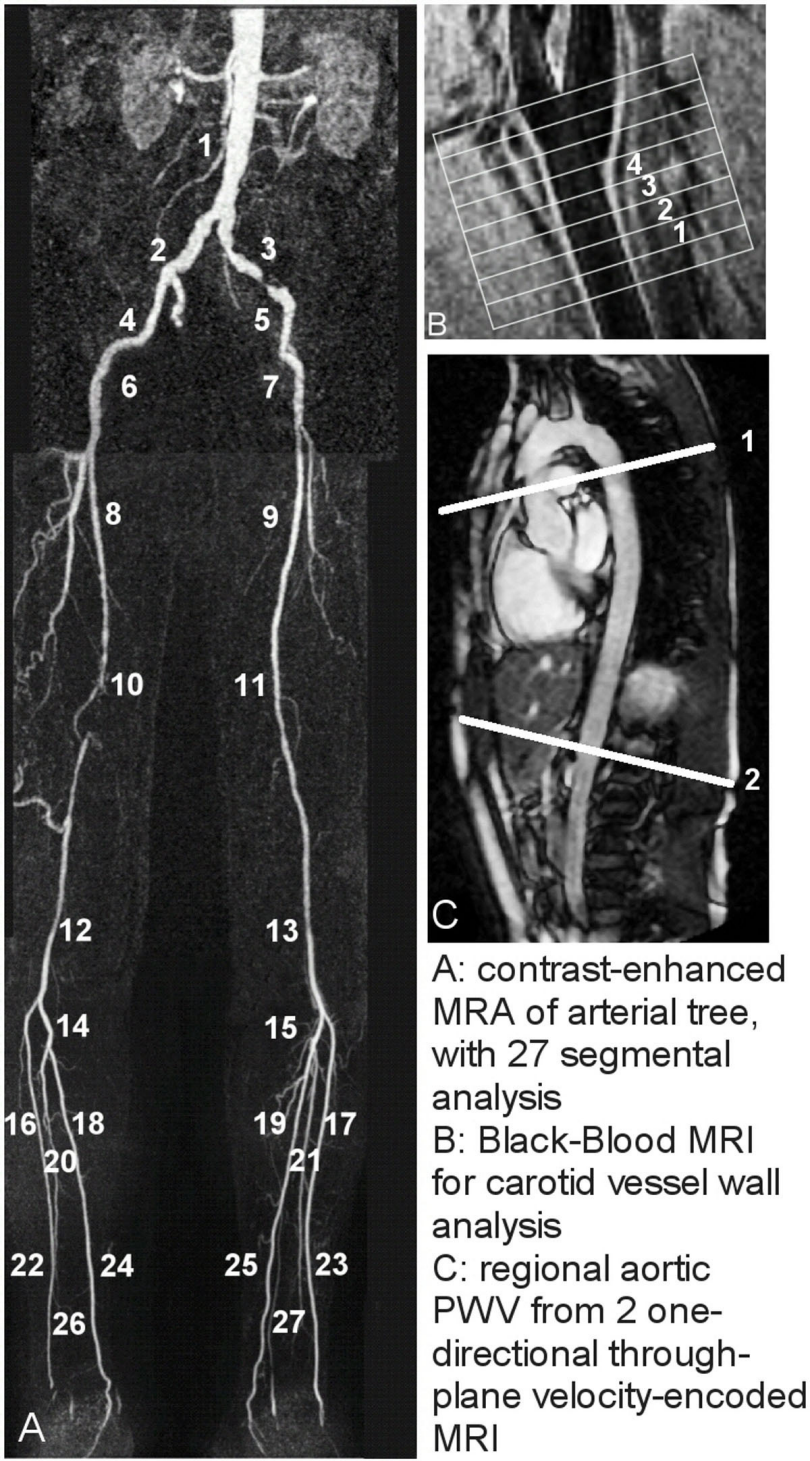


Forty-two patients (23 men; mean age 64±10years) with clinically suspected peripheral arterial occlusive disease (PAOD) were included. Standardized single-injection 3-station moving-table CE-MRA was performed at 3T MRI (Philips) with 0.2mmol/kg body weight Dotarem (Guerbet). Carotid VWT and aortic PWV were assessed during the same examination. With CE-MRA, the peripheral arteries were divided into 27 segments (Figure 1A). Visual stenosis classification was performed per segment in consensus by two radiologists in blinded manner: class 1 (0%-stenosis), 2 (1-50%), 3 (51-75%), 4 (76-99%), 5 (100%). Mean stenosis class (MSC) averaged over 27 segments per patient was determined. Four transverse images of the left common carotid artery, obtained by multi-slice 2D dual inversion recovery BB fast gradient-echo (Figure 1B)were analyzed and mean vessel wall area (VWA) per slice was manually determined. PWV was assessed for the proximal (ascending to thoracic descending) aorta and distal (thoracic descending to abdominal) aorta by applying two one-directional through-plane VE MRI acquisitions with high temporal resolution (Figure 1C). PWV was obtained from systolic wave propagation analysis based on the transit-time method. Regional aortic PWV was compared with carotid VWA indexed for body surface area (BSA) and mean peripheral stenosis class.

## Results

Mean Fontaine class was 2.3±0.6. Average of MSC per patient presented on CE-MRA was 1.6±0.4 (range: 1.0 to 3.6). Spearman correlation between MSC and proximal aorta PWV was 0.19 (p=0.25), for distal aorta PWV 0.74 (p<0.001) and for carotid VWA/BSA 0.53 (p<0.001). Linear regression models for regional aortic PWV and carotid VWA/BSA as dependent variables and age and MSC as predictors are constructed and presented in Table 1.

**Table 1:** Linear regression modeling between proximal and distal aortic PWV and carotid VWA/BSA as dependent variables and age and mean peripheral stenosis class (MSC) as predictors

		PWVproximal	PWVdistal	VWA/BSA
	R	0.45	0.63	0.57

Age	β	0.50	0.39	0.40

	β	0.10	0.02	0.02

MSC	β	-0.11	0.33	0.24

	β	0.56	0.04	0.15

## Conclusions

In patients with PAOD, mean peripheral stenosis severity is well-correlated with PWV in the descending to abdominal aorta and to a lesser extent with the carotid vessel wall thickness, while correlation with PWV in the proximal aorta is absent. These findings remain after correction for age, indicating site-specific coupling between aortic wall stiffness and atherosclerotic burden.

## Funding

None

